# Supplementary material for: A blueprint for precise and fault-tolerant analog neural networks
Source: Nat Commun. 2024 Jun 14;15:5098. doi: 10.1038/s41467-024-49324-8 (PMC11178814; doi:10.1038/s41467-024-49324-8)
Supplement: Supplementary file 3 — Author Checklist [file 41467_2024_49324_MOESM3_ESM.pdf]

nature portfolio

Author Checklist

NComms-23-42979A

Please check the items below carefully and add a response in each row of the table to indicate the changes that you have made. Please also check through any additional marked-up edits we may have provided within the manuscript file.

Abstract and editor's summary

Our guidance:	Your response:
Please shorten the abstract to 150 words or fewer.	We shortened the abstract to be <150 words.

Author information

We ask that you consult with your coauthors to ensure that all names, affiliations, and titles are represented correctly. Note that if any authors are added or removed after this point then all authors will be requested to provide approval documentation that could potentially delay the production of your paper.	We confirm that all author names, affiliations, and titles are represented correctly.

Article structure

Our guidance:	Your response:
We can accommodate up to 10 display items (Figures or Tables) in the main article. Each Figure and Table must fit easily within an A4 page (210 x 297 mm). Please ensure that the number and size of your Figures and Tables fulfill these requirements to avoid any delay in the acceptance of your article.	We reduced the number of display items down to 10. We provide all figures in PDF format and we confirm that all figure sizes are compatible with the requirements.
**Please ensure your main manuscript file includes the following sections, in this order:**<br>*Title*<br>*Author list*<br>*Affiliations*<br>*Abstract*<br>*Introduction*<br>*Results*<br>*Discussion (optional)*<br>*Results and Discussion (optional)*<br>*Methods*<br>*Data Availability*<br>*Code Availability (if relevant)*<br>*References*	We confirm that the manuscript file includes the sections listed on the left in the provided order.

Acknowledgments<br>*Author Contributions Statement*<br>*Competing Interests Statement*<br>*Tables*<br>*Figure Legends/Captions (for main text figures)*<br>We do not edit Supplementary Information files; they will be uploaded with the published article as they are submitted with the final version of your manuscript. Any tracked changes should be removed from the file and the file should be provided as a PDF file. Supplementary Figures do not need to be provided separately.	

Main text

Our guidance:	Your response:
We allow only one level of subheadings in the Results section. Please remove secondary subheadings.	We removed all the secondary subheadings in the main text.
Please do not use italics, bold font, underlining or speech marks/quotation marks except in headings unless required for technical terms (in both the main text and the display items).	We confirm that no bold or italic font, no underlined text, and no speech/quotation marks are used in the manuscript.
Please make sure that mathematical terms throughout your manuscript and Supplementary Information (including in figures, figure axes, and legends) conform strictly to the following guidelines. Equations must be supplied in editable format, and not as images. Scalar variables (e.g. x, V, χ) must be typeset in italic, whereas multi-letter variables and functions (e.g. log) must be formatted in roman. Vectors (such as the wavevector k or the magnetic field vector B) must be typeset in bold without italics.	We made sure all mathematical terms follow the rules stated on the left.

Figures and Tables

Our guidance:	Your response:
Please see the guidelines linked below for detailed instructions about how your figures should be prepared. Following these instructions will reduce the chances of delays should we need to request replacement artwork from you at a later stage.<br><br>https://www.nature.com/documents/NRJs-guide-to-preparing-final-artwork.pdf	We confirm that our figures comply with the provided guidelines.
Please ensure that data presented in a plot, chart or other visual representation format shows data distribution clearly (e.g. dot plots, box-and-whisker plots, violin plots). When using bar charts, please overlay the corresponding data points (as dot plots) whenever possible and always for n ≤ 10. All box-plot elements (center line, limits, whiskers, points) should be defined in the legends accompanied by precise n numbers.<br>**Please note that data presentation has to be revised to comply with our policy in figure(s) xxx**	We stated the sample size (the number of replications of the experiment) in the plots where we show a data distribution (only Fig. 2a). We added the box element definitions to the legend of this figure. The rest of the charts are provided as bar plots or dot/line plots where the experiments are deterministic and were run only once so there are no error bars provided.
Wherever statistics have been derived (e.g. error bars, box plots, statistical significance) the legend needs to provide and define the n number (i.e. the sample size used to derive statistics) as a precise value (not a range). Please define how many replicates were performed and whether they are biological (derived from different experimental units or subjects) or technical (multiple contemporary measurements from the same experimental unit or subject). Samples should be unambiguously described, including a clear definition of the unit of study. In studies using model organisms, cell lines, primary cell cultures, plants or micro-organisms, the unit of study is the smallest object that could be randomly and independently assigned to an intervention. The groups being compared, including control groups, should be clearly defined. If no control group has been used, the rationale for this should be stated. Please ensure there are enough details about sample collection to distinguish between independent data points and technical replicates- splitting a	We confirm that we define the sample sizes as a precise value. Our experiments are not biological.

biological sample into 3 tubes or wells receiving the same treatment does not constitute independent replication.<br><br>We strongly discourage deriving statistics from technical replicates or less than 3 biological replicates, unless there is a clear scientific justification for why providing this information is important. Conflating technical and biological variability, e.g., by pooling technically replicates samples across independent experiments is strongly discouraged.<br>**Please note that this information is missing in the legend(s) of figure(s) xxx**<br><br>**Please provide a precise value of 'n' in the legend(s) of figure(s) xxx**<br><br>**Although 'n' is provided, please describe the nature of entity for 'n' in the legend(s) of figure(s) xxx**<br><br>**The sample size 'n' indicated in the legend(s) does not match the data points plotted in the graph. Please ensure the sample size is accurately described in the legend(s) of figure(s) xxx**	
We strongly discourage deriving statistics from technical replicates or less than 3 biological replicates, unless there is a clear scientific justification for why providing this information is important. Conflating technical and biological variability, e.g., by pooling technical replicate samples across independent experiments is strongly discouraged. (For examples of expected description of statistics in figure legends, please see the following https://www.nature.com/articles/s41467-019-11636-5).<br>Statistics such as error bars, sigificance and p-values, cannot be derived from n<3 and must be removed from all such cases.<br><br>**Please note that this should be rectified for figure(s) xxx**	N/A
All error bars need to be defined in the legends (e.g. SD, SEM) together with a measure of centre (e.g. mean, median). For example, the legends should state something along the lines of "Data are presented as mean values +/- SEM" as appropriate. All box plots need to be defined in the legends in terms of minima, maxima, centre, bounds of box and whiskers and percentile.<br>**Please note that the error bars/error bands need to be defined in the legend(s) of figure(s) xxx**<br><br>**Please note that the measure of centre for the error bars/error bands needs to be defined in the legend(s) of figure(s) xxx**<br><br>**Please note that the box plots need to be defined in terms of minima, maxima, centre, bounds of box and whiskers and percentile in the legend(s) of figure(s) xxx**	We added the explanation for the box elements and whiskers in the legend of Fig 2a.
The figure legends must indicate the statistical test used. Where appropriate, please indicate in the figure legends whether the statistical tests were one-sided or two-sided and whether adjustments were made for multiple comparisons. For null hypothesis testing, please indicate the test statistic (e.g. F, t, r) with confidence intervals, effect sizes, degrees of freedom and P values noted. Please provide the test results (e.g. P values) as exact values whenever possible and with confidence intervals noted.<br>**Please indicate the statistical test used for data analysis and where appropriate, please specify whether it was one-sided or two-sided and whether adjustments were made for multiple comparisons, in the legend(s) of figure(s) xxx**<br><br>**Please note that the information on whether the statistical test used was one-sided or two-sided, where appropriate, is missing in the legend(s) of figure(s) xxx**<br><br>**Please note that the exact p value should be provided, when possible, in the legend(s) of figure(s) xxx**<br><br>**Please indicate what */ **/ ***/ **** represents; if this represents p value(s), please indicate the statistical test used and where appropriate, specify whether it was one-sided or two-sided and whether adjustments were made for multiple comparisons and the exact p value in the**	N/A

**legend(s) of figure(s) xxx**<br><br>**Please note that for the figure(s) xxx, */ **/ ***/ **** p-value(s) and statistical test(s) are indicated in the legend(s); however, comparison for the same has not been represented in the figure(s). Please rectify this in the figure(s) or legend(s) as applicable. Also, if appropriate, please provide exact p-value(s).**	
**Please revise this information in the legend(s) of figure(s) xxx**	
Please ensure that all blots and gels are accompanied by the locations of molecular weight/size markers. Where it is necessary to crop blots, please ensure that at least one marker position is present.<br><br>Please supply uncropped and unprocessed scans of the most important blots in the Source Data file or as a supplementary figure in the Supplementary Information. This should be cited once in the Methods section. For an example of presentation of full scan blots, see the Source Data file of https://www.nature.com/articles/s41467-020-16984-1#Sec35 and for more information, please refer to https://www.nature.com/nature-research/editorial-policies/image-integrity.<br><br>Quantitative comparisons between samples on different gels/blots are discouraged; if this is unavoidable, the figure legend must state that the samples derive from the same experiment and that gels/blots were processed in parallel.<br><br>Vertically sliced images that juxtapose lanes that were non-adjacent in the gel must have a clear separation or a black line delineating the boundary between the gels. Loading controls (e.g. GAPDH, actin) must be run on the same blot.<br><br>Sample processing controls run on different gels must be identified as such in the figure legends, and distinctly from loading controls.<br>**Please note that the units of molecular weight markers are missing for figure(s) xxx**<br><br>**Figure(s) xxx require(s) molecular weight markers**<br><br>**Figure(s) xxx require(s) uncropped and unprocessed scans**	N/A
Please ensure that all micrographs include a scale bar and this scale bar is defined on the panels or in the figure legends.<br>**Please note that scale bar is missing for figure(s) xxx**<br><br>**Please note that the scale bar needs to be defined for figure(s) xxx**	**N/A**
Test Additional Comments	
Tables must be editable and prepared using the table menu in Word, the table environment of LaTeX or the table functionality of ChemDraw where relevant.	Our tables were prepared in LaTex and are editable.
Shadings or symbols in graphs must be defined in some fashion. We prefer that you use a key within the image; do not include colored symbols in the legend/caption.	We confirm that the colors and symbols are defined within the figures.
Any abbreviations, symbols or colours present in your figures must be defined in the associated legends.	We confirm that all abbreviations in figures are defined in figure legends.
Your manuscript includes more than 10 display items (figures and/or tables). Please either merge figures together or move some of the display items into the Supplementary Information. Ensure that all display items are labelled and cited correctly and describe merged figures with panel labels (a, b, c, etc.). Please be aware that to enable typesetting of papers, the number of display items should be proportionate with the word length - those with word counts less than 2,000 should have no more than 4 display items.	We reduced the number of display items to 10.

Data and Code

Our guidance:	Your response:

Nature journals strongly support public availability of data and code. Please deposit the data and code used in your paper into a public data repository, or alternatively, present the data as Supplementary Information. If data can only be shared on request, please explain why in your Data Availability Statement, and also in the correspondence with your editor.<br><br>Please note that for some data types, deposition in a public repository is mandatory. Any restrictions on sharing of these data types must be clearly indicated in the statement and discussed with the editor. More information on our data deposition policies and available repositories can be found here:<br>https://www.nature.com/nature-research/editorial-policies/reporting-standards#availability-of-data	The code is proprietary to a company (Lightmatter) and can be shared upon reasonable request.
All published manuscripts reporting original research in Nature Portfolio journals must include a data availability statement, within the Methods and under the heading 'Data Availability'.<br><br>The data availability statement must make the conditions of access to the “minimum dataset” that are necessary to interpret, verify and extend the research in the article, transparent to readers. We ask that you don’t use phrases like ‘available on reasonable request’ but instead specify any restrictions to accessing your data as described below.<br><br>This minimum dataset may be provided through deposition in public community/discipline-specific repositories, custom proprietary repositories or general repositories like Figshare, Zenodo and Dryad. Providing large datasets in supplementary information is strongly discouraged and the preferred approach is to make data available in repositories. Please see https://www.springernature.com/gp/authors/research-data-policy/recommended-repositories for a list of recommended repositories.<br><br>If DOIs are provided, we also strongly encourage including these in the Reference list (authors, title, publisher (repository name), identifier, year).<br><br>The Data Availability Statement should also reference any source data published alongside the paper.<br><br>For clinical datasets or third party data, please ensure that the Data Availability statement adheres to our policy (https://www.nature.com/nature-research/editorial-policies/reporting-standards#availability-of-data)<br><br>If data are unavailable, please indicate the exact reasons why data cannot be made available in a suitable public repository or upon request, including any conditions related to ethical approval, consent from study subjects, commercial or legal restrictions, etc.<br><br>For data that are available under restricted access, the Data Availability statement must specify<br>- the reasons for access restrictions<br>- what the restrictions are<br>- how one can get access to the data<br>- who to contact to request access<br>- any restrictions on who the data can be made available to or for which purpose<br>- the expected timeframe for response to access requests<br>- for how long the data will be available once access has been granted.	The data are proprietary to a company (Lightmatter) and can be shared upon reasonable request.
Please use the following template to provide all the information stated above:<br><br>The XX data generated in this study have been deposited in the YY database under accession code ZZ [add hyperlink here]. The XX data are available under restricted access for {insert reason}, access can be obtained by {explain how}. The raw XX data are protected and are not available due to data privacy laws. The processed XX data are available at YY. The XX data generated in this study are provided in the Supplementary Information/Source Data file. The XX data used in this study are available in the YY database under accession code ZZ [Add hyperlink here].	We used the provided template in the code and data availability statements.

Methods

Our guidance:	Your response:
Sufficient details of the experiments must be provided in the Methods section such that they could be reproduced without reference to published papers. Use of the term "as described previously" is not encouraged.	We confirm that the experiments can be reproduced by using the information in the Methods section.

End matter

Our guidance:	Your response:
The Competing Interests statement must encompass all authors. Please edit the statement accordingly (such as by adding 'The remaining authors declare no competing interests', if this is accurate).	We modified the competing interests statement to encompass all authors.
Nature Portfolio defines Competing Interest (CI) as financial and non-financial interests (including but not limited to funding, employment, stocks, shares, patents, personal or professional relationships with individuals or institutions, and unpaid membership advocacy) that could be perceived to directly undermine the objectivity, integrity, and value of a publication, or could be seen as having an influence on the judgments and actions of authors with regard to objective data presentation, analysis, and interpretation.<br><br>Please thoroughly review our policy on Competing Interests and include a detailed statement both in your final manuscript file and in our manuscript tracking system. Please ensure the statements are identical in both. Be specific about how each point stated relates to the research and list applicable author initials, and/or patent numbers.<br><br>If there are no competing interests, a negative statement must be included.<br><br>https://www.nature.com/nature-research/editorial-policies/competing-interests	Updated.
Please confirm that all relevant funding awarded to each author is described in the Acknowledgements section. List each grant number, followed by the initials of the author who received it.	We confirm that all relevant funding is listed in the Acknowledgements section.

Preparing your manuscript files

Our guidance:	Your response:
Unless otherwise stated please limit individual file sizes to approximately 30MB. We strongly encourage the use of repositories for large datasets or source data due to size considerations.	We confirm that all files are under 30 MB.
Please supply a brief (maximum 250 characters, including spaces) summary of the main findings of the paper to be used on our website and in our e-alerts. The summary should be written in the third person in language suitable for a broad audience. The summary may be edited by the editors prior to publication. Please provide this summary in your cover letter.	This study explores the use of the residue number system to overcome precision challenges in analog computing, paving the way for unleashing its full potential as next-generation AI hardware for advanced tasks. (also included in the cover letter)
To ensure maximum visibility for your work, we may post about your paper following publication. If you would like us to include the X (formerly Twitter) handles of the first author(s), corresponding author(s), lab or institution in this post, please provide them in your cover letter. We would also welcome your suggestions for hashtags to use when posting about the work.	We provided the corresponding social media handles in our cover letter.
Please supply the main manuscript file in either Microsoft Word or LaTeX format	We provide our manuscript in LaTeX.
Please provide figures as individual vector files with editable text. Acceptable file types for figures are .ai, .eps, .pdf, .ppt or Chem Draw for fully editable vector-based art. For detailed guidance on figure preparation, see https://www.nature.com/documents/aj-artworkguidelines.pdf	We provide all figures in .pdf format
The use or adaptation of previously published images is strongly discouraged. If this is unavoidable, please request the necessary rights documentation to re-use such material from the relevant copyright holders and return this to us when you submit your revised manuscript. Please check whether your manuscript or Supplementary Information contain third-party images, such as	We confirm that the figures in our manuscript are not from previously published images.

figures from the literature, stock photos, clip art or commercial satellite and map data.<br><br>For more information on what constitutes ownership by a third party, please contact our Editorial Assistant at naturecommunications@nature.com	

Forms to complete

Our guidance:	Your response:
**Editorial Policy Checklist**<br>Please update and upload a final version of the Editorial Policy Checklist with your revised manuscript files. A blank Editorial Policy Checklist can be found via the link below. Note that this form is a dynamic 'smart pdf' and must be downloaded and completed in Adobe Reader.<br>Please update your current checklist or download from:<br>https://www.nature.com/documents/nr-editorial-policy-checklist.zip	We revised and reuploaded the Editorial Policy Checklist.
**Reporting Summary**<br>Please revise the Reporting Summary according to the requests below. After making the requested changes, please be sure to include the final version of your Reporting Summary in your submission as a supplementary information file. Please note that this form is a dynamic 'smart pdf' and must therefore be downloaded and completed in Adobe Reader, instead of opening it in a web browser.<br>Please update your current checklist or download from:<br>https://www.nature.com/documents/nr-reporting-summary.pdf	We revised and reuploaded the Reporting Summary.

Reporting Summary

Our guidance:	Your response:

You will need to upload:

Editorial Policy Checklist	✓
Completed Third Party Rights Table (if relevant)	N/A
A point-by-point response to the reviewers' comments	N/A
A completed copy of this checklist	✓
The main manuscript file in either Microsoft Word or LaTeX format	✓
Separate Figure files	✓
ChemDraw files	N/A
